# High‐Power Surgical Laser for Treatment of Oral Manifestations of Mucopolysaccharidosis VI: A Case Report

**DOI:** 10.1111/scd.70224

**Published:** 2026-08-08

**Authors:** Regina Maria Raffaele, Mario Eduardo Baldo, Bianca Ribeiro Marques, Stefan Gabriel Gonçalves Martiniano, Gleyson Kleber do Amaral Silva, Rafael Ferreira

**Affiliations:** ^1^ Department of Pediatric Dentistry and Hospital Dentistry Campo Grande MS Brazil; ^2^ Department of Health and Development, UFMS Dom Bosco Catholic University ‐ Mato Grosso do Sul Campo Grande MS Brazil; ^3^ Multidisciplinary Program – Critical Patient Care Federal University of Mato Grosso do Sul Campo Grande MS Brazil; ^4^ Department of Stomatology, FOP/UNICAMP Federal University of Mato Grosso do Sul (FAODO/UFMS) Campo Grande MS Brazil; ^5^ Department of Dentistry Federal University of Mato Grosso do Sul Campo Grande MS Brazil

## Abstract

Mucopolysaccharidosis type VI (MPS VI), a rare metabolic disorder, results from glycosaminoglycan accumulation in tissues, frequently leading to hyperplastic gingival alterations that necessitate comprehensive intervention. An 11‐year‐old male patient diagnosed with MPS VI required hospitalization due to complications associated with a ventriculoperitoneal shunt (VPS) valve. A dental evaluation identified bone malformation in the maxillomandibular complex accompanied by pronounced gingival hyperplasia. The surgical team performed a gingivoplasty in the operating room under general anesthesia, employing a combined technique involving manual scalpels and a diode laser to excise hyperplastic gingival tissue and cauterize bleeding sites. The patient exhibited an uneventful postoperative recovery, with no hemorrhagic or infectious complications. The diode laser demonstrated safety and efficacy in removing gingival tissue in this patient with MPS VI, facilitating local tissue repair, enhancing the aesthetic and functional profile, and ultimately improving the patient's oral health and quality of life.

## 1 Introduction

Mucopolysaccharidosis (MPS) is a group of rare genetic diseases resulting from the deficiency of lysosomal enzymes responsible for the degradation of glycosaminoglycans (GAGs), leading to their accumulation in cells and tissues. The pathological deposition of GAGs leads to dysfunction and progressive cell death, resulting in the impairment of most organs [1]. Divided into seven different types: I, III, IV, VI, VII, and IX, MPS has a low prevalence, ranging from 1.04 to 4.8 cases per 100,000 live births, and can vary significantly depending on the ethnic profile of individuals [2]. Specifically, MPS type VI, also known as Maroteaux–Lamy syndrome, is described as the form with the most compromised skeletal phenotype. Although neurological function is usually preserved, these patients may have severe limitations due to hearing impairment, visual deficit, and physical impairment [1].

The most important radiographic manifestations of dental abnormalities observed in these individuals with MPS are the presence of supernumerary teeth, conoid teeth, taurodontism, impacted teeth, and root laceration. Enlarged dental follicles are also common in these individuals and may develop into cystic formations. Bone abnormalities associated with MPS include a higher frequency of bone rarefaction, condylar hypoplasia, and radiolucent bone lesions, as well as shortening of the mandibular ramus [3, 4].

Gingival hyperplasia is reported as the main soft tissue manifestation of the syndrome, associated with an anterior open bite, macroglossia, and a high ogival palate [5, 6, 7]. Depending on the degree of impairment of the stomatognathic system, surgical interventions may be necessary, mainly to correct or alleviate dental dysfunctions and improve their functionality [3].

The purpose of this report is to describe the importance of a dental approach in a hospital setting, highlighting the feasibility of using procedures and resources such as a laser scalpel in the operating room, especially to join forces with the multidisciplinary approach and improve the quality of life of a patient with MPS type VI

### 1.1 Case Report

An 11‐year‐old male patient with a previous diagnosis of Mucopolysaccharidosis was admitted to ABCG Santa Casa Hospital in Campo Grande – MS due to complications associated with a ventriculoperitoneal shunt valve (VPS). According to his medical history, MPS was diagnosed at the age of 3, based on clinical characteristics compatible with the syndrome and the results of genetic tests. Since then, the patient has undergone multidisciplinary follow‐up, with regular visits to a pediatric cardiologist, pulmonologist, geneticist, otorhinolaryngologist, pediatric neurologist, and neuro‐ophthalmologist.

At age four, the patient was started on the beta‐blocker carvedilol to control mitral regurgitation and underwent placement of a ventriculoperitoneal shunt (VPS) to drain excess cerebrospinal fluid. In 2024, the patient presented to his physician with headache and vomiting. Upon investigation, a CT scan revealed the presence of a brain abscess related to the previously implanted valve, which required surgical removal.

Physically, the patient presented with characteristic signs of MPS, including hyperextension of the cervical spine, thoracolumbar scoliosis, mitral insufficiency, restriction of joint movement, brachydactyly, umbilical hernia, facial changes such as bitemporal retraction, low nasal root, bilateral ocular proptosis, horizontal nystagmus, hearing loss, and adenoid hyperplasia. Considering that enzyme replacement therapy (ERT) is less effective for severe bone manifestations and is not capable of correcting cognitive impairment, this approach was not applicable to the patient. Due to recurrent upper respiratory tract infections and predominant mouth breathing, the patient had previously undergone adenoidectomy and tonsillectomy for marked adenoid hyperplasia.

**FIGURE 1:**
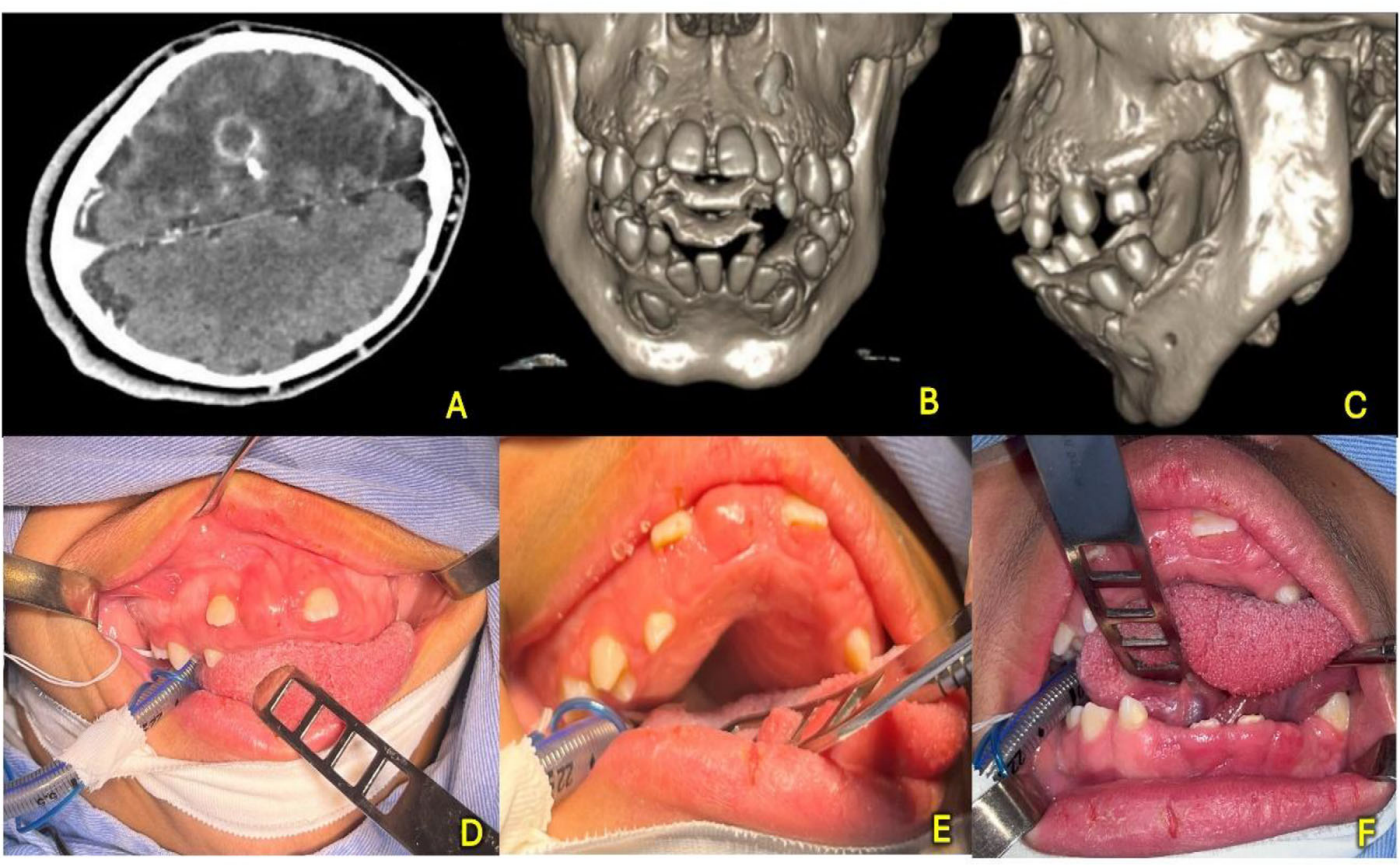
Tomographic and clinical images of the patient: (A) sagittal section; 3D reconstruction with (B) frontal section and (C) sagittal section; intra‐oral clinical images: (D) frontal view; (E) occlusal view of the upper arch; (F) lower arch. *Source*: the authors.

The in‐patient dental assessment revealed significant malocclusion (Angle Class II with no lip seal), anterior open bite with permanent and deciduous teeth positioned in a fan shape (Figure 1). There was also diffuse hyperplastic gingival growth, an ogival palate, macroglossia, included teeth, and a consequent delay in their eruption.

During the anamnesis, the mother reported that she had noticed changes in the gums as early as infancy. She also informed us that the only medications she took continuously were Carvedilol 25 mg a day and Furosemide 40 mg a day; according to her, the patient was not taking anticonvulsants and had no significant nutritional deficit.

Due to the extent of the procedures, the dental team decided to perform the procedures under general anesthesia. For the surgical procedure, the pharmacological protocol and airway management were planned based on the patient's weight (45 kg). Initially, midazolam was administered intravenously at a dose of 2 mg. The primary anesthetic induction was with propofol at a dose of 100 mg (approximately 2.2 mg/kg), ensuring rapid loss of consciousness. Assisted orotracheal intubation was performed, allowing the patient to remain perfectly stable and monitored until the start of the surgical procedure. Even under sedation, local infiltration anesthesia was used with mepivacaine hydrochloride 20 mg/ml associated with epinephrine 10 µg/ml. To perform gingivoplasty and reshape the gingival and papillary contour, manual scalpels and a high‐power diode laser were used.

**FIGURE 2:**
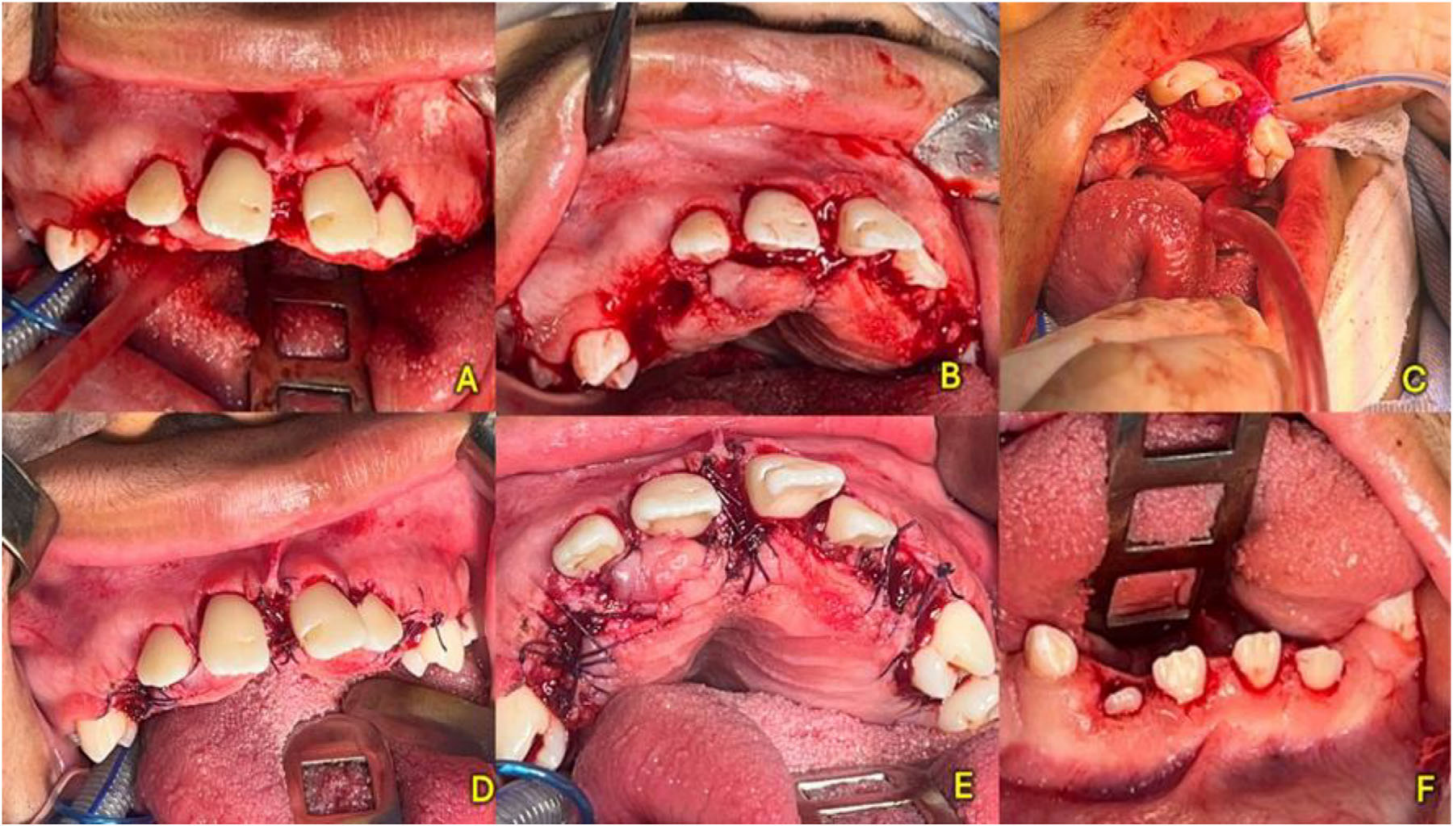
‐ Clinical images of the trans‐operative period: (A) frontal view; (B) incisal view; (C) application of the surgical laser; (D) final clinical aspect; (E) sutures; (F) final clinical aspect of the lower arch. *Source*: the authors.

The beam of choice was considered due to its high capacity for simultaneous cutting and coagulation, resulting in low bleeding, less postoperative pain, and excellent healing. A high‐power diode laser with a wavelength of 980 ± 5 nm (Soft Lase Pro Zap Laser, Pleasant Hill, California, USA), with a power output of 1.2 W in gated mode (0,5 ms), using a fiber diameter of 400 µm, and a pulse fluency of 20 Hz with an energy density of 90J/cm2, followed by irrigation with normal saline solution. During the removal of the gingival tissue, mobility was observed in the upper deciduous canines, which were removed during the surgical stage. After gingivoplasty (Figure 2), coronal root scraping and smoothing were performed, followed by cleaning of the operated area with 0.12% chlorhexidine digluconate aqueous solution.

**FIGURE 3:**
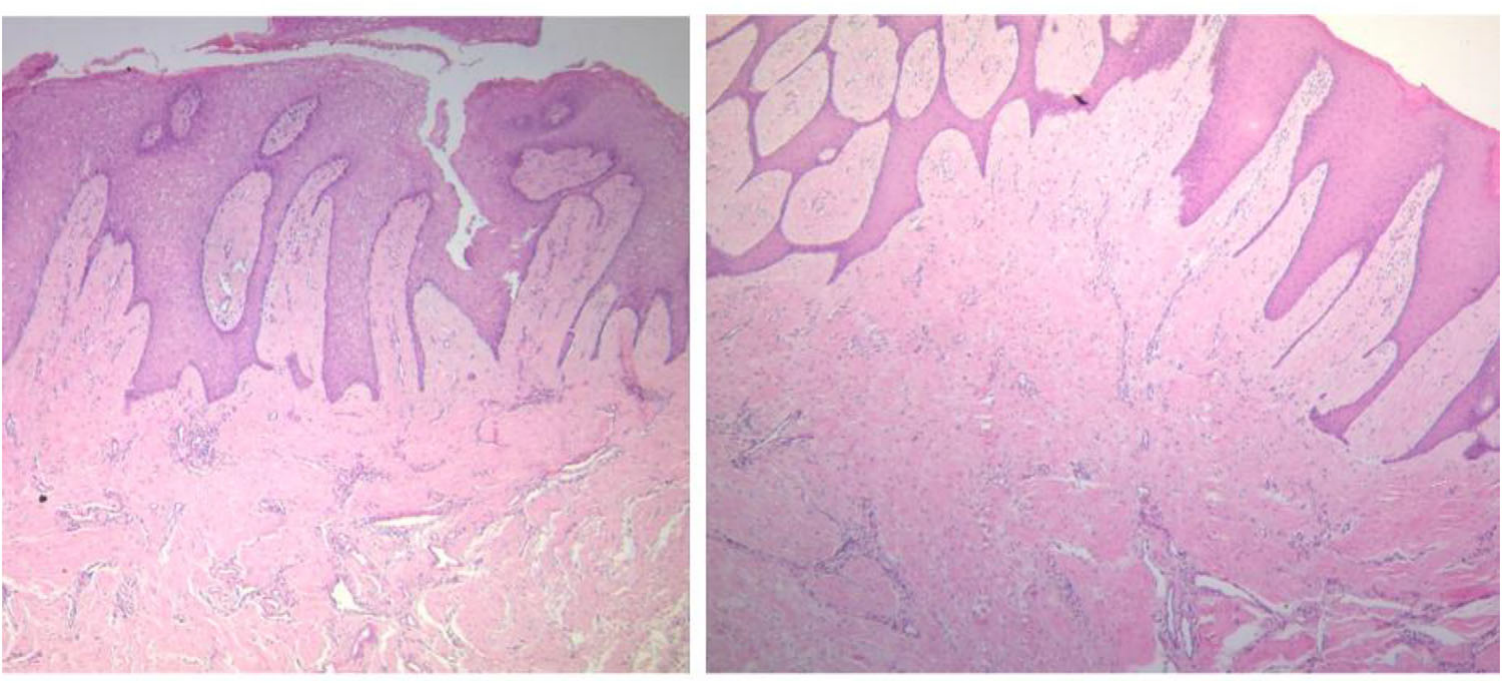
Microscopic examination revealing mucosal fragments lined by hyperkeratinized stratified squamous epithelium with elongated epithelial ridge projections (acanthosis). The spinous layer appears thickened, with epithelial cells displaying vacuolated morphology and pale cytoplasm, a finding consistent with intracytoplasmic accumulation of glycosaminoglycans, characteristic of mucopolysaccharidoses. Within the subepithelial connective tissue, increased deposition of collagen fibers is observed, arranged in dense and irregular bundles imparting a myxoid appearance to the stroma, with associated rarefaction of elastic fibers.

The dental surgeon employed 4.0‐monofilament nylon thread to suture the surgical margins and prescribed prophylactic antibiotic therapy and analgesic medication for the patient. The surgical team collected gingival tissue fragments for histopathological analysis (Figure 3). In the postoperative guidelines, the dental team instructed the patient and their caregivers to perform careful oral hygiene, utilizing an ultra‐soft toothbrush immersed in a 0.12% chlorhexidine digluconate solution twice daily for 14 consecutive days.

The patient was hospitalized for 72 h under the supervision of the hospital dental team, who closely monitored his postoperative condition. Following discharge, the same team carried out weekly outpatient evaluations. At the one‐month postoperative assessment, the patient demonstrated marked clinical improvement, with satisfactory wound healing and maintenance of periodontal tissue homeostasis. During subsequent follow‐up visits, the patient's mother reported a notable improvement in masticatory function, describing enhanced chewing efficiency during meals. She further noted that the process was well tolerated and pain‐free, attributed to the establishment of proper occlusal contact.

## 2 Discussion

The systemic features associated with MPS VI include developmental delay, skeletal dysplasias, joint limitations, heart disease, hepatosplenomegaly, umbilical hernias, dysmorphic facial features, macrocephaly, recurrent upper respiratory tract infections, hearing loss, and eye structures involvement [8, 9]. The age of diagnosis varies between the different types of mucopolysaccharidosis, correlating with the severity of the clinical manifestations. The most severe forms, such as MPS VI, can be identified early, possibly due to the early presentation of the first clinical signs and symptoms [1]. Genetic counseling should be encouraged in suspected cases, since early diagnosis and a multidisciplinary approach are essential for effectively managing these patients [10].

In a common way, growth begins to slow from 18 months of age and stops by the end of childhood. The stature is short, with a short neck and trunk, and abnormal spinal undergrowth, but the sternum grows normally, giving a “pigeon chest” appearance [11, 12]. These characteristics can be challenging when performing orotracheal intubation maneuvers during procedures under sedation or general anesthesia, and pose a high risk in these patients. This is due to anatomical variations such as a relatively short neck, a large tongue (lingual hypotonia), and protrusion [4]

Excision of soft tissues with lasers provides efficient hemostasis, reduction of the inflammatory process, and analgesia, which facilitates the maintenance of proper oral hygiene and contributes to the long‐term success of periodontal treatment [13]. High‐power lasers are absorbed by hemoglobin, although poorly absorbed by water in the interstitial space. It is worth noting that the light beam can penetrate approximately 10 mm into the tissue, promoting a phenomenon known as soft tissue photocoagulation, which significantly reduces gingival bleeding and allows safe and efficient removal of gingival tissue [14]. Clinical practice endorsed surgical laser procedures for bactericidal and local hemostatic effects, combined with better visibility of the surgical field and a reduction in recurrent hyperplasia. It is worth noting that laser removal of mucosal tissue also reduces the average duration of the procedure, minimizes postoperative discomfort, and may lead to a more favorable healing profile, in addition to reducing local edema [13].

The dental team's involvement proves essential in evaluating patients with alterations to the facial morpho‐skeletal profile. A multidisciplinary approach, fostering collaboration among healthcare professionals from various specialties, remains critical. Incorporating a dental surgeon promotes preventive measures and clinical strategies that enhance the patient's quality of life ([4, 15].

## 3 Conclusion

The dental treatment of a patient with MPS VI, conducted in the operating room using a diode laser to remove hyperplastic gingiva, proved to be an effective and safe procedure. It yielded satisfactory outcomes in terms of bleeding control and postoperative recovery. This case highlights the value of employing advanced technologies to manage oral health issues in patients with rare metabolic disorders.

Furthermore, the inclusion of a dental surgeon in multidisciplinary teams is crucial, not only for delivering specialized clinical care but also for providing tailored guidance on oral health management for patients with MPS.

## Funding

This research received no specific grant from any funding agency in the public, commercial, or not‐for‐profit sectors

## Consent

The patient's legal representative expressed her agreement to the use all data generated by her husband's care procedures for research purposes by signing a consent form. Therefore, the author(s) declare compliance with ethical principles in research, exams, and procedures used as background to this case report.

## Conflicts of Interest

The author(s) declared no potential conflicts of interest with respect to the research, authorship, and/or publication of this article.
